# DeepHeteroCDA: circRNA–drug sensitivity associations prediction via multi-scale heterogeneous network and graph attention mechanism

**DOI:** 10.1093/bib/bbaf159

**Published:** 2025-04-14

**Authors:** Zhijian Huang, Kai Chen, Xiaojun Xiao, Ziyu Fan, Yuanpeng Zhang, Lei Deng

**Affiliations:** School of Computer Science and Engineering, Central South University, No. 932, South Lushan Road, Changsha 410083, Hunan, China; School of Computer Science and Engineering, Central South University, No. 932, South Lushan Road, Changsha 410083, Hunan, China; School of Software, Xinjiang University, No. 666, Shengli Road, Urumqi 830046, Xinjiang, China; School of Computer Science and Engineering, Central South University, No. 932, South Lushan Road, Changsha 410083, Hunan, China; School of Software, Xinjiang University, No. 666, Shengli Road, Urumqi 830046, Xinjiang, China; School of Computer Science and Engineering, Central South University, No. 932, South Lushan Road, Changsha 410083, Hunan, China

**Keywords:** circRNA–drug sensitivity associations, heterogeneous graph, deep learning, graph attention mechanism

## Abstract

Drug sensitivity is essential for identifying effective treatments. Meanwhile, circular RNA (circRNA) has potential in disease research and therapy. Uncovering the associations between circRNAs and cellular drug sensitivity is crucial for understanding drug response and resistance mechanisms. In this study, we proposed DeepHeteroCDA, a novel circRNA–drug sensitivity association prediction method based on multi-scale heterogeneous network and graph attention mechanism. We first constructed a heterogeneous graph based on drug–drug similarity, circRNA–circRNA similarity, and known circRNA–drug sensitivity associations. Then, we embedded the 2D structure of drugs into the circRNA–drug sensitivity heterogeneous graph and use graph convolutional networks (GCN) to extract fine-grained embeddings of drug. Finally, by simultaneously updating graph attention network for processing heterogeneous networks and GCN for processing drug structures, we constructed a multi-scale heterogeneous network and use a fully connected layer to predict the circRNA–drug sensitivity associations. Extensive experimental results highlight the superior of DeepHeteroCDA. The visualization experiment shows that DeepHeteroCDA can effectively extract the association information. The case studies demonstrated the effectiveness of our model in identifying potential circRNA–drug sensitivity associations. The source code and dataset are available at https://github.com/Hhhzj-7/DeepHeteroCDA.

## Introduction

Circular RNAs (circRNAs) constitute a distinct category of endogenous non-coding RNAs, mainly produced by back-splicing or lariat-driven processes occurring within genes [1]. In contrast to linear RNAs that feature 5’ caps and 3’ poly(A) tails, circRNAs create covalently closed loops, devoid of both 5’ to 3’ polarity and polyadenylation [1]. This unique structure not only renders circRNAs resistant to exonuclease-mediated degradation, thereby providing them with significantly enhanced stability compared to linear RNAs [2], but also contributes to their ubiquity across various cell types and organisms. As research into circRNAs advances, their broad biological significance and potential functions are becoming increasingly recognized, attracting widespread attention in the scientific community. For instance, certain circRNAs contain miRNA response elements (MREs), allowing them to function as miRNA sponges, a mechanism that regulates miRNA activity and downstream gene expression [3]. MiRNAs, $\sim $22 nucleotides in length, play a key role in post-transcriptional regulation by binding to 3’-untranslated regions (3’-UTRs) of target mRNAs, leading to mRNA destabilization and translational repression [4]. Moreover, circRNAs such as CircHIPK3, Circ_0006528, and Circ_0004870 have been linked to cancer-related hallmarks, promoting tumorigenesis by modulating tumor suppressors, apoptotic pathways, and cellular replication [5]. Due to their stability, conservation, ubiquity, and specificity, circRNAs are emerging as highly promising prognostic and diagnostic biomarkers in cancer research [6].

Drug resistance continues to be a major obstacle in cancer treatment, and recent studies have highlighted the crucial role of circRNAs in regulating this resistance [7]. For example, circRNA-circ_0076305 has been demonstrated to promote resistance to cisplatin (DDP) in non-small cell lung cancer (NSCLC) by regulating ABCC1 through miR-186-5p, thereby reducing the efficacy of the drug in NSCLC treatment [8]. In glioma, circ_0072083 has been implicated in temozolomide (TMZ) resistance by enhancing NANOG expression through multiple pathways, including regulation by miR-1252-5p and ALKBH5-mediated demethylation [9]. Moreover, CircNR3C1 regulates the BRD4/C-myc complex in bladder cancer (BC). CircNR3C1 interacts with BRD4, disrupting the BRD4/C-myc complex that promotes BC progression, and in vivo studies showed that ectopic expression of C-myc can partially reverse the tumor-suppressive effects of circNR3C1 [10]. These findings shed light on the complex mechanisms of drug resistance involving circRNAs. However, our understanding of the complex interactions between circRNAs and drug sensitivity is still limited.

Identifying associations between circRNAs and drug sensitivity using experimental methods is frequently hindered by high costs, intensive labor, and time-consuming processes.

Nevertheless, numerous advanced deep learning methods have been developed for link prediction [11–15]. By reformulating the circRNA–drug sensitivity associations prediction as a link prediction task, deep learning techniques can also be employed to address this challenge. Deng *et al*. [16] introduced GATECDA, a framework based on a graph attention auto-encoder, which utilizes graph attention mechanisms to effectively predict circRNA–drug sensitivity associations by capturing essential information from sparse, high-dimensional data. Similarly, Yang *et al*. [17] developed MNGACDA, leveraging multimodal networks that incorporate graph auto-encoders and attention mechanisms to predict these associations. Luo *et al*. [18] proposed DPMGCDA, a framework that employs dual perspective learning and a path-masked graph autoencoder to predict circRNA–drug sensitivity associations. Although existing methods have made certain progress, they lack the extraction of the intrinsic features of drug molecules and fail to consider the differences in information weights among different nodes in the networks. This leads to a reduced capacity of the model to capture the information of drug molecule nodes, resulting in the insufficient consideration of crucial structural information and node features within the network.

Here, we introduce DeepHeteroCDA, an innovative deep learning framework that utilizes multi-scale heterogeneous networks for predicting circRNA–drug sensitivity associations. The approach starts by organizing circRNA host gene sequences, drug structural information, and associations between circRNAs and drugs. A heterogeneous graph is then constructed based on drug–drug and circRNA–circRNA similarity and known circRNA–drug sensitivity associations. We employ a Graph Attention Network (GAT) [19] to update the representations of heterogeneous nodes with adaptive weights, thereby enhancing the extraction of network-level information. To mine the molecular-level information of chemical structure of drugs, the model represents the drug as a 2D structure, with atoms as nodes and chemical bonds as edges, and utilizes GCN [20] to compute drug embedding vectors. Therefore, we constructed a multi-scale heterogeneous graph, in which the atomic-level 2D topological features of drugs were also adaptively updated during the feature update process of the circRNA–drug network. Compared to the circRNA–drug networks proposed by other methods, our multi-scale heterogeneous networks can further capture the fine-grained structural features of small molecules, enabling the model to better explore the complex interactions between drugs and circRNAs. Finally, a dense neural network is used to predict potential associations.

To evaluate the effectiveness of DeepHeteroCDA, we conducted a comprehensive assessment using the benchmark dataset, comparing our proposed method with the state-of-the-art methods. The results highlight the superior predictive performance of DeepHeteroCDA. Additionally, an ablation study is performed to evaluate the performance of each component. The case study involving three specific drugs further demonstrates the practical utility of DeepHeteroCDA and the predicted results can aid conventional experiments by enabling the preselection of effective circRNA–drug sensitivity associations.

## Materials and methods

### Data sets

From the circRic database [21], following a stringent filtering process, associations with a false discovery rate exceeding 0.05 were excluded. The resulting benchmark dataset comprises 4134 experimentally validated circRNA–drug sensitivity associations, involving 271 unique circRNAs and 218 distinct drugs. Based on key associations, a bipartite circRNA–drug topology network is constructed, denoted ${AM} \in \mathbb{R}^{271 \times 218}$. If an experimental association between the *p*th circRNA and the *q*th drug is verified, the corresponding entry $AM_{pq}$ is assigned a value of 1. Additionally, 4134 unverified circRNA–drug associations were randomly selected as negative samples, making up just 6.99% of all potential circRNA–drug pairs, thereby representing a relatively small portion. Therefore, the probability that genuine interactions are misclassified as negative samples is low. Following this selection, we curated a comprehensive dataset of 8268 circRNA–drug pairs, ensuring a balanced dataset by maintaining an equal number of positive and negative samples. The SMILES [22] representations of the drugs were retrieved from the DrugBank database [23].

### Overview of DeepHeteroCDA

**Figure 1:**
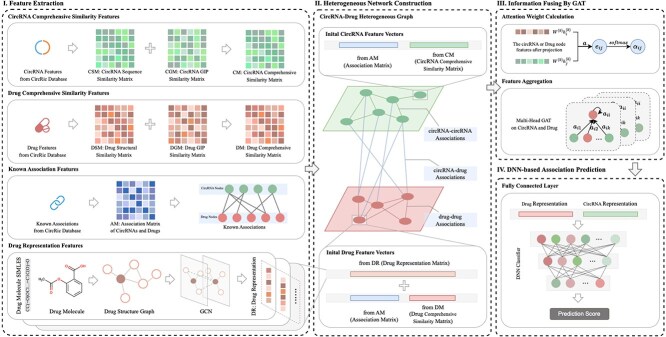
The framework of DeepHeteroCDA. (I) Feature extraction: extracts similarity features for circRNA and drugs, including sequence, structure, and known association data. GCN is used to aggregate information from neighboring atoms and chemical bonds through graph convolutional layers, learning the structural features of molecules. (II) Heterogeneous network construction: builds a network integrating circRNA and drug interactions. (III) Information fusing by GAT: uses graph attention networks to aggregate node features. GAT leverage attention mechanisms to weigh and aggregate features of circRNAs and drugs based on their relevance. (IV) DNN-based association prediction: predicts circRNA-drug associations using a deep neural network.

To predict potential circRNA–drug sensitivity associations, we present DeepHeteroCDA, an innovative method based on a multi-scale heterogeneous network and attention mechanism. Our approach employs an inductive method to extract topological information of drugs, which is subsequently combined with circRNA–drug heterogeneous network. The workflow of the DeepHeteroCDA model, as illustrated in Fig. 1, consists of the following five key steps:

Step 1: CircRNA and drug similarity measurement. CircRNA similarity is quantified using two metrics: sequence-based similarity and GIP (Gaussian interaction profile) kernel similarity [24, 25]. Similarly, drug similarity is evaluated from two perspectives: structural similarity and GIP kernel similarity. The comprehensive similarity matrices for circRNAs and drugs are obtained by merging the respective similarity matrices.

Step 2: Construction of a circRNA–drug heterogeneous graph. A heterogeneous graph is constructed, wherein the similar neighbors for each circRNA or drug are retained to capture essential relationships. The features of circRNA and drug nodes are then mapped into a shared vector space, enabling a unified representation that facilitates the integration of multiple data modalities.

Step 3: Drug topology information extraction. In this step, drug nodes are transformed to 2D graph, where atoms are treated as nodes and chemical bonds as edges. GCNs are then employed to extract comprehensive and informative representations of drugs from these graph-based structures. Combining the drug topology graph with the circRNA–drug heterogeneous graph, we achieve a multi-scale heterogeneous network.

Step 4: Aggregation of central node information. GAT is utilized to aggregate information from neighboring nodes, with distinct attention weights assigned to each neighbor. This adaptive attention mechanism allows the model to prioritize more informative nodes during the aggregation process, thereby capturing nuanced relationships between the central node and its neighbors.

Step 5: CircRNA–drug associations prediction. We concatenated the final circRNA and drug features from step 4. This representation is passed through a fully connected layer to generate a prediction score, with cross-entropy loss used for optimization.

The subsequent sections will offer an in-depth analysis and explanation of each of the outlined steps.

#### CircRNA similarity measurement

As described in [17], we quantify circRNA similarity by calculating the sequence similarity between their host genes. This calculation is based on the Levenshtein distance between sequences, using the ratio function. This method produces an adjacency matrix, denoted $CSM \in R^{271 \times 271}$, which stores the sequence similarity data of the host genes.

Meanwhile, it is significant to recognize that the adjacency matrix $CSM$ is sparse. To achieve comprehensive information mining of circRNA similarity, we employ the GIP kernel similarity, which has proven effective in evaluating similarity across various biological entities. We calculate the GIP kernel similarity for circRNAs based on the circRNA–drug sensitivity association matrix $AM$, under the assumption that circRNAs associated with the same drug sensitivity are more likely to be similar. The resulting GIP kernel similarity matrix of circRNA is denoted as $CGM \in R^{271 \times 271}$. Inspired by Xiao *et al*. [26], we can derive the circRNA similarity as follows:


(1) \begin{align*}& CM_{i j}=\left\{\begin{array}{ll}\frac{\left(CSM_{i j}+CGM_{i j}\right)}{2}, & \text{ if}\ CSM_{i j} \neq 0 \\CGM_{i j}, & \text{ otherwise }\end{array}\right.,\end{align*}

where $CM_{ij}$ is the final similarity of circRNA $i$ and $j$, $CSM_{ij}$ is their sequence similarity, and $CGM_{ij}$ is their GIP kernel similarity. Since $CSM_{ij}$ and $CGM_{ij}$ capture similarity at different levels, the final similarity is computed as their average when $CSM_{i j} \neq 0$ to integrate their complementary information. However, the adjacency matrix $CSM$ is sparse. When $CSM_{i j} = 0$, the final similarity is derived solely from $CGM_{ij}$ as the final similarity measure to avoid the potential bias caused by the lack of information from $CSM_{i j}$.

#### Drug similarity measurement

Drug similarity can be evaluated from two perspectives. First, given the significant influence of drug structure on its function, the drug similarity is assessed based on structural characteristics. Drug structure data are obtained from the PubChem database, and RDKit is used to compute the topological fingerprint for each drug. The structural similarity between drugs is then calculated using the Tanimoto coefficient, resulting in the drug structure similarity matrix, denoted as $DSM \in R^{218 \times 218}$. Simultaneously, the GIP kernel similarity matrix for drugs, denoted as $DGM \in R^{218 \times 218}$, is calculated.

Similar to circRNA, the drug similarity is derived by combining the two aforementioned similarity measures:


(2) \begin{align*}& DM_{mn} =\left\{\begin{array}{ll}\frac{\left(DSM_{mn}+DGM_{mn} \right)}{2}, & \text{ if } DSM_{mn} \neq 0 \\DGM_{mn}, & \text{ otherwise }\end{array}\right.,\end{align*}

where $DM_{mn}$ is the final similarity of drug $m$ and $n$, $DSM_{mn}$ is their sequence similarity and $DGM_{mn}$ is their GIP kernel similarity.

#### GCN for drug representation

To transform the SMILES of drugs into molecular graphs, we utilize the RDKit toolkit, which enables atoms to be modeled as nodes and bonds as edges in the graph. The initial features of nodes are computed using MoleculeNet [27]. The core of the framework is the implementation of GCN, specifically designed for drug feature extraction. This method allows for the effective extraction of pertinent knowledge from the intricate topological features of drugs inherent to drugs.

The GCN is a neural network architecture specifically designed to process data structured in the form of graphs. In graph-based data, the adjacency matrix typically represents the relationships between nodes. GCN functions by generating lower-dimensional node representations through an iterative process, utilizing multiple layers to capture the structural information of the graph.

The following outlines the iterative procedure:


(3) \begin{align*} & \hat{A} = A + I_{N}, \end{align*}

(4) \begin{align*} & H^{l+1}= \sigma \left ( D^{-\frac{1}{2}}\hat{A}D^{-\frac{1}{2}}H^{l}W^{l} \right ), \end{align*}

where $I_{N}$ denoting the identity matrix, $A$ representing the adjacency matrix, and $\hat{A}$ is the adjacency matrix of the undirected graph with added self-connections. $H^{l}$ refers to the embeddings at the $l$th layer, $D$ is the degree matrix of $A$, $W^{l}$ is a layer-specific trainable parameter, $H^{0}$ is the initial node feature matrix, and $\sigma (\cdot )$ represents ReLU [28].

To capture the overall structural information of the graph in a global feature representation, max pooling is used to aggregate the learned node embeddings into a graph-level feature vector. This operation ensures that local node-level information is effectively summarized at the graph level. Specifically, for drug molecular graphs, after the GCN has learned the embeddings for each atom, global max pooling is applied to derive a single feature representation for each drug. This process can be mathematically represented as:


(5) \begin{align*}& DR_{d} = \max_{o=1}^{n_{d}} H_{d}^{L}[o,:],\end{align*}

where $DR_{d}$ denotes the global feature vector of the $d$-th drug, $H_{d}^{L}[o,:]$ represents the feature vector of the $o$th node in the $d$th drug’s molecular graph at the $L$th layer of the GCN, $n_{d}$ is the number of atoms in the molecular graph of the $d$th drug. By applying max pooling, we select the maximum value from the feature vectors of all nodes to obtain a global representation for the drug.

Subsequently, the global feature vectors for all drugs are concatenated into a complete drug representation matrix $DC$, represented as:


(6) \begin{align*}& DR = \begin{bmatrix} DR_{1} \\ DR_{2} \\ \vdots \\ DR_{N} \end{bmatrix},\end{align*}

where $DR \in \mathbb{R}^{218 \times 489}$, with 218 representing the number of drugs and 489 being the dimensionality of the feature vectors learned by the GCN. Through the global max pooling operation, the node-level representations of drug molecular graphs are aggregated into drug-level global feature representations, which are subsequently used for enhancing the representation of drugs in heterogeneous networks.

#### Heterogeneous graph construction

To achieve circRNA–drug association prediction, we construct a heterogeneous network comprising 271 circRNAs and 218 drugs. The heterogeneous network integrates two types of edges: interaction edges and similarity edges. The interaction edges originate from the circRNA–drug associations, representing exclusive connections between circRNA and drug pairs. However, the bipartite nature of this graph restricts its ability to effectively aggregate information across the network. To address this limitation, we enhance the bipartite graph by incorporating additional similarity networks, capturing the relationships between circRNA pairs and drug pairs. Since similarity edges in the circRNA–circRNA and drug–drug networks used to form dense connections, which can introduce noise and complicate the model. To mitigate this, we retain only the eight most similar neighbors for each circRNA or drug, thereby simplifying the graph while preserving critical similarity relationships. Let $CM$ represents the similarity matrix of circRNA nodes, $DM$ denotes the similarity matrix of drug nodes, and $AM$ signifies the association matrix of circRNAs and drugs. We can define the heterogeneous network as follows:


(7) \begin{align*}& HG = \begin{bmatrix} CM & AM \\ AM^{T} & DM \end{bmatrix}.\end{align*}

The construction of the heterogeneous graph is essential for enriching the information flow across circRNA and drug nodes. By incorporating interaction and similarity relationships, the model can better capture the intricate patterns and associations that exist between circRNAs and drugs, thereby enhancing predictive performance.

#### GAT on heterogeneous graphs

Given that the heterogeneous network contains two distinct types of nodes, the initial representation dimensions of the nodes differ. Aligning their features to the same dimensional space ensures that the aggregation process is coherent and compatible. The initial circRNA node features, denoted as $X_{c}$, are represented as follows:


(8) \begin{align*}& X_{c} = \begin{bmatrix} AM || CM \end{bmatrix},\end{align*}

where $\left | \right |$ represents the concatenation.

In contrast to the circRNA nodes, which simply concatenate adjacency and similarity matrices to form their initial feature set, the drug nodes undergo an additional layer of processing to capture structural and relational complexities. Specifically, the initial drug features, denoted as $X_{d}$, are constructed by first concatenating the adjacency matrix $AM^{T}$ with $DM$, followed by adding the drug representation matrix $DR$. This representation goes beyond conventional feature vectors by embedding intricate graph-based descriptions of the drug, significantly enhancing the node’s capacity to retain chemical structure and similarity information. The initial drug features can be expressed as:


(9) \begin{align*}& X_{d} = \left( AM^{T} \, || \, DM \right) + DR.\end{align*}

Next, we project the circRNA and drug features into the same dimensional space. We apply two transformation matrices, $W_{c} \in \mathbb{R}^{256 \times 271}$ for circRNAs and $W_{d} \in \mathbb{R}^{256 \times 218}$ for drugs, where “256” denotes the shared feature dimension. The transformation employs the ReLU nonlinearity to adjust the feature dimensions. The steps for projecting circRNA and drug nodes are outlined below:


(10) \begin{align*} & H_{c}= W_{c} X_{c}, \end{align*}

(11) \begin{align*} & H_{d}= W_{d} X_{d}, \end{align*}

where $W_{c}$ and $W_{d}$ are learnable parameters for mapping the 489-dimensional circRNA node features into a reduced 256-dimensional space. In this context, $X_{c}$ refers to the initial feature set of the circRNA, and $H_{c}$ is the features after projection. Similarly, $W_{d}$ is the linear transformation matrix applied to the drug nodes, which also have 489-dimensional features that are reduced to 256 dimensions. The drug nodes’ original features are denoted by $X_{d}$, and the corresponding projected features are represented by $H_{d}$.

GAT utilizes a muti-head attention mechanism to extract the features of each node’s neighborhood by layering multiple network levels, assigning varying weights to the neighboring nodes based on their relative importance. The input to the GAT consists of the graph’s structural relationships and the individual node attributes, resulting in a new set of node embeddings as output. For instance, considering nodes $a$ and $b$, GAT applies a linear transformation to both nodes individually to derive higher level node representations. Subsequently, a self-attention mechanism is applied to node pairs to calculate the weights $e_{ab}$, reflecting the influence of node $b$ on node $a$. The computation of the weights can be expressed using the following formula:


(12) \begin{align*}& e_{ab} = \sigma \left ( a^{k} \left [ W^{k} h_{a}^{k} \parallel W^{k} h_{b}^{k} \right] \right ),\end{align*}

where $\sigma $ denotes the LeakyReLU. To ensure comparability among the weights of node $a$’s adjacent nodes, we further apply softmax normalization to adjust the weights of the target node and its surrounding neighbors. After acquiring the normalized attention coefficients, we compute the linear combination of the corresponding neighbor features. Finally, the output feature vector is derived by applying a nonlinear activation function.

#### Deep neural network layer

To predict the interaction probability $\hat{P_{cd}}$ between circRNA $c$ and drug $d$, we concatenate the final feature vectors of the circRNA and drug nodes derived from the GAT. These concatenated features are then passed through a fully connected layer to compute the predicted probability $\hat{P_{cd}}$, as follows:


(13) \begin{align*}& \hat{P_{cd}} = \text{sigmoid}\left( \text{ReLU}\left( W \left( h_{c}) \parallel h_{d} \right) \right) \right).\end{align*}

Subsequently, the model’s performance is evaluated by calculating the loss between the predicted probability and the actual label using the cross-entropy loss function.

## Results

### Evaluation metrics

To ensure the reliability and stability of DeepHeteroCDA, we perform five-fold cross-validation (5-CV) and 10-fold cross-validation (10-CV) to evaluate the performance of DeepHeteroCDA. In this experiment, we introduced the area under receiver operating characteristic (ROC) curve (AUC), the area under precision-recall (PR) curve (AUPR), accuracy (ACC), F1-Score (F1), and recall (R) as assessment metrics for evaluating the performance of DeepHeteroCDA and comparison methods. Their definition is as follows:


(14) \begin{align*} & \mathrm{TPR} = \frac{TP}{TP + FN}, \quad \mathrm{FPR} = \frac{FP}{TP + FN}, \end{align*}

(15) \begin{align*} & \mathrm{Accuracy} = \frac{TP + TN}{TP + TN + FP + FN}, \end{align*}

(16) \begin{align*} & \mathrm{Precision} = \frac{TP}{TP + FP}, \quad \mathrm{Recall} = \frac{TP}{TP + FN}, \end{align*}

(17) \begin{align*} & \mathrm{F1\text{-}score} = 2 \times \frac{\mathrm{Precision} \cdot \mathrm{Recall}}{\mathrm{Precision} + \mathrm{Recall}}, \end{align*}

where $TP$ and $TN$ denote true positive and true negative instances, respectively, while $FP$ and $FN$ represent false positive and false negative instances. AUC is the area under the ROC curve that measures a model’s ability to distinguish between positive and negative classes across all thresholds, with a higher value indicating better performance. AUPR is the area under the precision–recall curve that evaluates a model’s ability to handle imbalanced data by focusing on positive class predictions, with a higher value indicating better performance.

### Comparison with other methods

To the best of our knowledge, there are currently only three computational approaches for predicting potential associations between circRNAs and drug sensitivity, namely DPMGCDA, MNGACDA, and GATECDA. To thoroughly assess the performance of DeepHeteroCDA, we extend our comparison to include these three models as well as four additional models that focus on other association prediction tasks within the bioinformatics domain. Additionally, we incorporate several well-established machine learning models, such as Support Vector Machine (SVM) [29], Random Forest (RF) [30], k-Nearest Neighbors (KNN) [31], XGBoost [32], and AdaBoost [33], which are widely recognized for their robustness and strong performance in association prediction across various domains. Additional details regarding the baseline methods are provided in the Supplementary material.

As shown in Table 1, our proposed approach, DeepHeteroCDA, demonstrated state-of-the-art performance in the 5-CV experiments, achieving an AUC of 0.9233, which is relatively higher than the second-best method, MNGACDA, by 1.5%, followed by improvements of 2.4% over DPMGCDA, 2.6% over XGBoost, 2.8% over LAGCN, 4.0% over RF, 4.3% over AdaBoost, 4.4% over GATECDA, 5.2% over GCNMDA, 5.6% over VGAMF, 6.8% over SVM, 6.9% over KNN, and 7.0% over VGAE. Similarly, DeepHeteroCDA outperformed all other methods in terms of AUPR, with an average value of 0.9293, which represents a relative improvement of 1.3% over DPMGCDA, 1.6% over MNGACDA, 3.0% over LAGCN, 3.3% over XGBoost, 4.1% over GATECDA, 4.6% over AdaBoost and RF, 6.1% over GCNMDA and KNN, 6.4% over VGAE, 7.3% over VGAMF, and 8.7% over SVM. In addition to AUC and AUPR, DeepHeteroCDA outperformed the other methods across all other metrics, including recall, F1-score, and accuracy.These results highlight the robustness and effectiveness of DeepHeteroCDA compared to the other seven methods evaluated in this study.

**Table 1:** Comparison experiments under five-fold CV

**Methods**	**AUC**	**AUPR**	**F1-score**	**Accuracy**	**Recall**
SVM	0.8648	0.8547	0.8049	0.7928	0.8550
RF	0.8881	0.8885	0.8204	0.8165	0.8383
KNN	0.8642	0.8760	0.7926	0.7901	0.8020
XGBoost	0.8997	0.8996	0.8294	0.8252	0.8494
AdaBoost	0.8852	0.8888	0.8207	0.8155	0.8443
VGAE	0.8628	0.8730	0.7988	0.7892	0.8227
VGAMF	0.8740	0.8662	0.8176	0.8104	0.8437
GCNMDA	0.8778	0.8762	0.8198	0.8119	0.8428
GATECDA	0.8846	0.8929	0.8194	0.8168	0.8316
LAGCN	0.8982	0.9023	0.8285	0.8261	0.8403
MNGACDA	0.9098	0.9150	0.8413	0.8379	0.8592
DPMGCDA	0.9015	0.9173	0.8408	0.8424	0.8325
DeepHeteroCDA	**0.9233**	**0.9293**	**0.8561**	**0.8520**	**0.8807**

Note: The best performance for each metric is marked in bold, while the second-best performance is marked in underlined.

**Figure 2:**
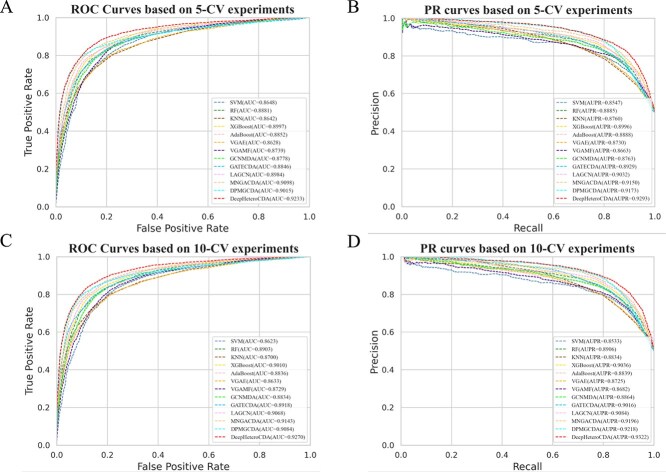
Comparison results of ROC curves and PR curves of DeepHeteroCDA with other methods under five-fold CV and 10-fold CV.

In the 10-fold cross-validation (10-CV), the DeepHeteroCDA method delivered the highest performance, achieving an AUC score of 0.9270. Comparing to the other methods, DeepHeteroCDA showed relative gains, with a 1.4% increase over MNGACDA, 2.1% over DPMGCDA, 2.3% over LAGCN, 2.9% over XGBoost, 3.9% over GATECDA, 4.1% over RF, 4.9% over AdaBoost and GCNMDA, 6.2% over VGAMF, 6.6% over KNN, 7.4% over VGAE, and 7.5% over SVM. Similarly, DeepHeteroCDA attained an average AUPR score of 0.9322, marking relative improvements of 1.1% over DPMGCDA, 1.4% over MNGACDA, 2.7% over LAGCN, 3.2% over XGBoost, 3.4% over GATECDA, 4.7% over RF, 5.2% over GCNMDA, 5.5% over AdaBoost and KNN, 6.8% over VGAE, 7.3% over VGAMF, and 9.2% over SVM. While DeepHeteroCDA achieved the highest accuracy and F1 score among all methods, it showed a slightly lower recall compared to LAGCN. However, the superior performance in terms of AUC, AUPR, and F1 score suggests that DeepHeteroCDA provides the most reliable predictive capabilities overall, as shown in Table 2. Notably, the overall performance in the 10-CV experiment slightly exceeded that of the 5-CV experiment. This enhancement is likely due to the larger dataset used in 10-CV, facilitating better model training and resulting in superior predictive outcomes. Fig. 2 displays the ROC and PR curves of DeepHeteroCDA and the comparison models under 5-CV and 10-CV. The results further demonstrate the superior performance of DeepHeteroCDA.

**Table 2:** Comparison experiments under 10-fold CV

**Methods**	**AUC**	**AUPR**	**F1-score**	**Accuracy**	**Recall**
SVM	0.8623	0.8533	0.8052	0.7977	0.8361
RF	0.8903	0.8906	0.8213	0.8176	0.8385
KNN	0.8700	0.8834	0.7970	0.7961	0.8005
XGBoost	0.9010	0.9036	0.8310	0.8273	0.8494
AdaBoost	0.8836	0.8839	0.8240	0.8198	0.8439
VGAE	0.8634	0.8725	0.7987	0.7865	0.8314
VGAMF	0.8729	0.8682	0.8113	0.8030	0.8471
GCNMDA	0.8834	0.8864	0.8225	0.8183	0.8420
GATECDA	0.8919	0.9016	0.8234	0.8211	0.8343
LAGCN	0.9065	0.9076	0.8425	0.8372	**0.8708**
MNGACDA	0.9143	0.9196	0.8445	0.8468	0.8324
DPMGCDA	0.9084	0.9218	0.8443	0.8458	0.8359
DeepHeteroCDA	**0.9270**	**0.9322**	**0.8570**	**0.8571**	0.8567

Note: The best performance for each metric is marked in bold, while the second-best performance is marked in underlined.

### Parameter sensitivity analysis

To evaluate the performance impact of six key hyperparameters: learning rate, dropout rate, hidden dimension of GAT, GAT layers, GAT heads and GCN layers, we conducted a series of experiments on the benchmark dataset under 5-CV by varying the values of six key hyperparameters within their respective ranges while keeping other settings constant.

**Figure 3:**
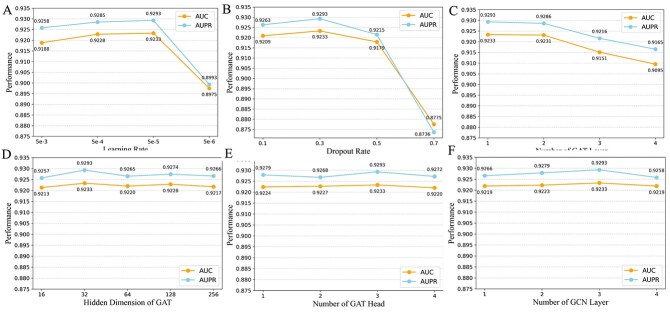
The influence of different hyperparameters on the model performance under 5-CV. (A) Learning rate; (B) dropout rate; (C) number of GAT layer; (D) hidden dimension of GAT; (E) number of GAT head; (F) number of GCN layer.

The learning rate determines how fast the model updates its weights during training. A well-tuned learning rate ensures stable and efficient convergence, avoiding overshooting and slow progress. We experimented with learning rates of 5e-3, 5e-4, 5e-5 and 5e-6. As shown in Fig. 3(A), the model performance steadily improved as the learning rate increased from 5e-3 to 5e-5. However, when the learning rate was further reduced to 5e-6, the performance dropped significantly, indicating that the rate was too small for the model to optimize its parameters effectively and reach optimal performance during training.

Dropout helps reduce overfitting by randomly deactivating neurons during training, encouraging the model to learn more robust features. We experimented with dropout rates of 0.1, 0.3, 0.5, and 0.7. As shown in Fig. 3(B), model performance significantly declined as the dropout rate increased. Excessive dropout caused the model to lose too much information, leading to unstable training and preventing effective learning. The optimal performance was achieved when the dropout rate was set to 0.3.

The number of GAT layers determines how many levels of node interactions are captured. Proper tuning of this parameter helps the model learn useful long-range dependencies without losing node-specific information. As shown in Fig. 3(C), the model achieves its best performance when the value is 1. The reason is that an excessively large number of GAT layers caused the model to overfit.

The hidden dimension of GAT defines the size of feature embeddings for each node. Tuning this hyperparameter optimizes the model’s expressiveness, allowing it to learn rich, meaningful node representations. As shown in Fig. 3(D), when the hidden dimension of GAT is 32, the predictive performance of DeepHeteroCDA reaches the best.

The number of GAT heads determines how many parallel attention mechanisms are used in the graph attention layers. Proper tuning of this hyperparameter helps the model capture diverse relationships between nodes from multiple perspectives. As shown in Fig. 3(E), the model achieves its best performance when the number of GAT heads is 3. This indicates that a suboptimal number of attention heads results in reduced performance, with the optimal number balancing between model complexity and the ability to capture sufficient information.

The number of GCN layers controls how many layers of convolutional operations are applied on the drugs, which influences the depth of information aggregation. Tuning this hyperparameter is essential for ensuring that the model captures both immediate neighborhood and multi-hop dependencies effectively. As shown in Fig. 3(F), the performance of DeepHeteroCDA reaches its peak when the number of GCN layers is 3. This suggests that deeper GCNs lead to better performance up to a certain point, after which increasing the number of layers may cause overfitting.

### Experimental analysis under different data conditions

The circRNA–drug sensitivity association scenarios under actual experimental conditions are often diverse. To ensure a comprehensive evaluation of our model’s robustness, we conducted experiments under various data conditions, including different training data sizes and data noise. Additionally, we performed a performance comparison with DPMGCDA.

To evaluate the model’s efficiency in leveraging data with limited circRNA–drug sensitivity associations, we first performed experiments using five-fold cross-validation with varying sizes of training data, where subsets of the training set (e.g. 90%, 80%, 70%, 60%) were randomly selected for training while the test set remained unchanged. As shown in the 4(A)-(C), the performance of both DeepHeteroCDA and DPMGCDA, as measured by AUC, AUPR, and F1 scores, declines as the size of training data decreases. Across all training data sizes, DeepHeteroCDA consistently outperforms DPMGCDA in all three metrics. The results demonstrate that DeepHeteroCDA maintains stable and superior performance across varying scales of training data.

**Figure 4:**
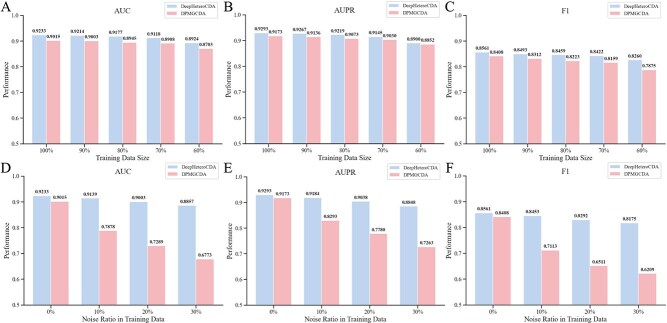
Performance comparison on three metrics under different data conditions. (A)-(C) are performance comparison on three metrics under different training data size. (D)-(E) are performance comparison on three metrics under different data noise.

Under real experimental conditions, data noise is often inevitable. To evaluate the models’ sensitivity to data quality under varying noise levels, we further tested the performance of models by altering a portion of unknown associations in the training set of the five-fold cross-validation to known associations (e.g. 10%, 20%, 30%). As shown in Fig. 4(D)-(F), when the data noise level increases from 0% to 30%, DeepHeteroCDA maintains relatively stable performance across all three metrics, while DPMGCDA exhibits a more significant decline. The reason may be that DPMGCDA relies entirely on the circRNA–drug network to learn association information. In contrast, our proposed DeepHeteroCDA not only leverages GAT to dynamically aggregate effective neighbor information but also employs GCN to learn fine-grained drug features independently of the association network. The results demonstrate that DeepHeteroCDA possesses strong generalization capabilities and robustness. To evaluate the performance of DeepHeteroCDA under different circRNA–drug sensitivity association scenarios, we further conducted additional experiments under different positive-negative sample ratios. The results can be found in Supplementary Table S1.

### Ablation study

To comprehensively evaluate the individual contributions of each component within DeepHeteroCDA, we have constructed several variants of DeepHeteroCDA. In contrast to prior methodologies, our DeepHeteroCDA model integrates the drug molecular graph in circRNA–drug heterogeneous networks and utilizes GCN to extract fine-grained drug-specific features.

Here are the specific meanings of each ablation variant:

- DeepHeteroCDA_w/o MG_ removes the step of mining topological graph features of drugs by GCN, and only used the structural similarity network and association network information of drugs to extract drug embeddings.
- DeepHeteroCDA_w/o GIP_ removes the GIP kernel similarity of circRNAs and drugs in the model.
- DeepHeteroCDA_w/o SG_ removes the sequence similarity of circRNAs and structure similarity of drugs in the model.
- DeepHeteroCDA_w/o GAT_ uses the circRNA and drug embeddings from the feature extraction module instead of the embeddings extracted by GAT.

In the conducted experiments, as presented in Table 3, the outcomes for DeepHeteroCDA_w/o GIP_ and DeepHeteroCDA_w/o SG_ exhibit lower performance compared to DeepHeteroCDA. This finding underscores the notion that the integration of diverse types of circRNA and drug features within DeepHeteroCDA yields superior results in comparison to incorporating only a single type of circRNA or drug feature. The performance of DeepHeteroCDA_w/o MG_ is notably inferior to that of DeepHeteroCDA, indicating the pivotal role of molecular structural features of drugs extracted by the GCN component. This observation underscores the importance of capturing the nuanced structural characteristics of drugs in enhancing predictive accuracy. Furthermore, the performance of DeepHeteroCDA_w/o GAT_ are also worse than DeepHeteroCDA’s performance. This outcome suggests that the integration of the GAT within DeepHeteroCDA holds value. The GAT module’s capability to assign variable weights to each edge, thereby emphasizing significant neighbors through enhanced weights, contributes to the overall performance boost observed in DeepHeteroCDA.

**Table 3:** Ablation studys under five-fold CV

**Methods**	**AUC**	**AUPR**	**F1-score**	**Accuracy**	**Recall**
DeepHeteroCDA_w/o GIP_	0.8920	0.8998	0.8198	0.8217	0.8068
DeepHeteroCDA_w/o SG_	0.9039	0.9102	0.8229	0.8288	0.7877
DeepHeteroCDA_w/o MG_	0.9132	0.9198	0.8399	0.8441	0.8119
DeepHeteroCDA_w/o GAT_	0.9129	0.9202	0.8378	0.8412	0.8102
DeepHeteroCDA	**0.9233**	**0.9293**	**0.8561**	**0.8520**	**0.8807**

### Visualization of DeepHeteroCDA embeddings

**Figure 5:**
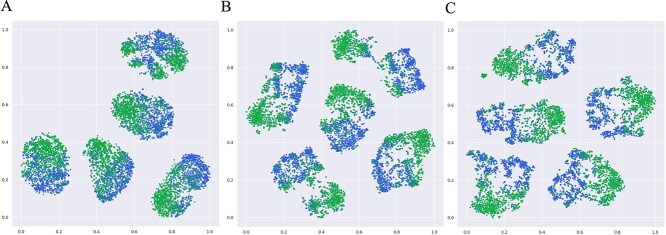
Visualization of DeepHeteroCDA embeddings across epochs in 5-CV. Two types of nodes represent whether circRNA and drug are associated in the concatenation embedding. (A) Epoch 1; (B) Epoch 20; (C) Epoch 200.

To further illustrate that DeepHeteroCDA can learn the association information between circRNA and drug sensitivity, we used t-SNE [34] to reduce the dimensionality and visualize the concatenation embedding of circRNA and drug from heterogenous network of DeepHeteroCDA. t-SNE is a dimensionality reduction technique used to visualize data in a lower-dimensional space. As shown in Fig. 5, the green points indicate an association between the circRNA and drug that form the concatenation embedding, while the blue points represent no association. As the number of training epochs increases, the green and blue points become more locally separated, with clearer boundaries between different classes and tighter clustering within the same class. The visualization results indicate that even without the prediction module, the embeddings derived from terogenous network of DeepHeteroCDA already capture rich association knowledge between circRNA and drugs.

### Case study

To assess DeepHeteroCDA’s capability in predicting novel associations between circRNAs and drugs, we conducted case study in the independent CTRP database [35]. We trained DeepHeteroCDA on our dataset and predicted the circRNA–drug sensitivity associations related to three drugs: Linifanib, Piperlongumine, and Vorinostat, in the independent CTRP database. The predicted results were ranked based on their predicted scores. The top 20 associations will be validated against the results in the CTRP database that have been experimentally confirmed.

For Linifanib [36], a multi-target VEGF and PDGFR receptor family inhibitor, the top 20 predicted circRNAs were evaluated. Among these, 17 circRNAs have been verified in the CTRP database, reinforcing the accuracy of DeepHeteroCDA’s predictions (see Table 4).

**Table 4:** The top 20 circRNAs associated with Linifanib

**Rank**	**CircRNA**	**Vertified**	**Rank**	**CircRNA**	**Vertified**
1	ANXA2^*^	$\bullet $	11	COL7A1^*^	$\bullet $
2	RIM1^*^	$\bullet $	12	PKM^*^	$\bullet $
3	CALD1^*^	$\bullet $	13	HSP90B1^*^	$\bullet $
4	VIM^*^	$\bullet $	14	LTBP3^*^	$\bullet $
5	LINC01089	$\circ $	15	COL8A1^*^	$\bullet $
6	COL6A2^*^	$\bullet $	16	KDELR1	$\circ $
7	DCBLD2^*^	$\bullet $	17	KRT7^*^	$\bullet $
8	FBLN1	$\circ $	18	MGAT4B^*^	$\bullet $
9	TAGLN2^*^	$\bullet $	19	HMGA2^*^	$\bullet $
10	CTTN^*^	$\bullet $	20	PYGB^*^	$\bullet $

‘$\bullet $’ indicates verified associations from CTRP, ‘$\circ $’ indicates non-significant associations. circRNAs marked with ‘^*^’ are verified.

Regarding Piperlongumine [37, 38], an alkaloid with recognized anti-tumor properties, the top 20 predicted circRNAs were also examined. Among these predicted results, 14 circRNAs have been corroborated by biological experiments in the CTRP database (see Table 5).

**Table 5:** The top 20 circRNAs associated with piperlongumine

**Rank**	**CircRNA**	**Vertified**	**Rank**	**CircRNA**	**Vertified**
1	COL3A1	$\circ $	11	LINC01089^*^	$\bullet $
2	BPTF	$\circ $	12	KRT7^*^	$\bullet $
3	EFEMP1^*^	$\bullet $	13	COL6A1^*^	$\bullet $
4	FBLN1^*^	$\bullet $	14	CALR	$\circ $
5	POLR2A^*^	$\bullet $	15	PTMS^*^	$\bullet $
6	ASPH^*^	$\bullet $	16	FLOT1^*^	$\bullet $
7	SERPINH1^*^	$\bullet $	17	CSRP1	$\circ $
8	LTBP3^*^	$\bullet $	18	MCAM	$\circ $
9	EFEMP2^*^	$\bullet $	19	PLCB3	$\circ $
10	FBN1^*^	$\bullet $	20	WASF1^*^	$\bullet $

circRNAs marked with ‘^*^’ are verified.

Lastly, for Vorinostat [39, 40], an HDAC inhibitor with anti-proliferative effects on various cancer cells, the top 20 predicted circRNAs were assessed. Among these predicted results, 15 circRNAs have been substantiated by experimental evidence in the CTRP database (see Table 6).

**Table 6:** The top 20 circRNAs associated with vorinostat

**Rank**	**CircRNA**	**Vertified**	**Rank**	**CircRNA**	**Vertified**
1	JUP	$\circ $	11	TFAP2A	$\circ $
2	ANP32B	$\bullet $	12	CXCL1	$\bullet $
3	NOP53	**-**	13	KDELR2	$\bullet $
4	PYGB	$\bullet $	14	HNRNPA2B1	$\circ $
5	TAGLN2	$\bullet $	15	KDELR1	$\bullet $
6	ARID1A	$\bullet $	16	SMC1A	$\bullet $
7	HSP90B1	$\circ $	17	CTSB	$\bullet $
8	PLOD1	$\bullet $	18	CALD1	$\bullet $
9	FLNB	$\bullet $	19	PRRC2A	$\bullet $
10	LGALS3BP	$\bullet $	20	FN1	$\bullet $

‘**-**’ indicates unconfirmed associations.

These case studies underscore the strong predictive ability of DeepHeteroCDA in identifying circRNA–drug associations, thereby emphasize its potential as a valuable tool in the realm of circRNA–drug sensitivity prediction. To improve the interpretability of our model, we further conducted SHAP (SHapley Additive exPlanations) [41] analysis on all circRNA–drug samples in our dataset to enhance the interpretability of our model. The results can be found in Supplementary Figs S2 and S3.

## Conclusions and discussion

In this work, we present a novel deep learning method namely DeepHeteroCDA for circRNA–drug sensitivity associations prediction. We initially constructed a heterogeneous network using drug–drug and circRNA–circRNA similarity along with known circRNA–drug sensitivity associations, and utilized GAT to dynamically model the intricate relationships among diverse nodes. By further representing drugs as 2D structures and utilizing GCN to mine structural features of drugs during the process of information extraction with GAT, we have achieved multi-scale information propagation in heterogeneous graph networks. Finally, we use a dense neural network to predict circRNA–drug sensitivity associations. Extensive experiments, including five-fold and 10-fold cross-validation, demonstrate that DeepHeteroCDA outperforms existing methods. Experiments under different data conditions further validate the robustness of DeepHeteroCDA. In case studies, the top-ranked circRNA predicted by DeepHeteroCDA show strong alignment with experimental evidence, further supporting its reliability. These results underscore DeepHeteroCDA’s potential as a powerful tool for advancing circRNA-related research and enhancing drug response predictions.

While the current study primarily focuses on predictive modeling, we recognize the importance of validating these predictions through experimental biology as part of our further work. We have outlined a workflow for integrating the model’s predictions with experimental biology. Specifically, our future research will focus on the following steps to bridge the gap between predictive modeling and experimental validation. First, we can use our model to rank significant circRNA–drug sensitivity instances to identify potential association. These predictions can then guide functional enrichment and pathway analyses to uncover potential mechanisms. Collaborating with experimental biologists, we can design targeted validation experiments based on our model’s prediction, including in vitro assays and in vivo studies. The experimental results can be used to iteratively refine our model, enhancing its accuracy and biological relevance. Finally, we can explore clinical applications, such as biomarkers for personalized medicine, leveraging the validated predictions from our model.

**Key Points**

- DeepHeteroCDA is a novel method that predict circRNA–drug sensitivity associations using multi-scale heterogeneous network and graph attention mechanism.
- We constructed a multi-scale heterogeneous network that can adaptively update the fine-grained features of drug molecules while mining the features of circRNAs and drugs in the heterogeneous network, enabling the model to better explore the complex interactions between circRNAs and drugs.
- The results of the comparative experiments highlight the superior predictive performance of DeepHeteroCDA. The case study underscore the strong predictive ability of DeepHeteroCDA in identifying potential circRNA–drug associations.
- DeepHeteroCDA is a useful bioinformatics tool that can assist biological experiments by filtering valid circRNA–drug sensitivity associations in advance.

## Supplementary Material

Supplementary_Materials_for_DeepHeteroCDA_bbaf159

## Data Availability

The source code and data sets are available at https://github.com/Hhhzj-7/DeepHeteroCDA.
