# Supplementary material for: DeepHeteroCDA: circRNA–drug sensitivity associations prediction via multi-scale heterogeneous network and graph attention mechanism
Source: Brief Bioinform. 2025 Apr 14;26(2):bbaf159. doi: 10.1093/bib/bbaf159 (PMC11995009; doi:10.1093/bib/bbaf159)
Supplement: Supplementary_Materials_for_DeepHeteroCDA_bbaf159 [file supplementary_materials_for_deepheterocda_bbaf159.pdf]

DeepHeteroCDA: circRNA-drug sensitivity associations prediction via multi-scale heterogeneous network and graph attention mechanism

Zhijian Huang [1], Kai Chen [1], Xiaojun Xiao [2], Ziyu Fan [1], Yuanpeng Zhang [2], Lei Deng [1,*]

*To whom correspondence should be addressed.

[1]School of Computer Science and Engineering, Central South University, Changsha, 410083, China and
[2]School of Software, Xinjiang University, Urumqi, 830046, China.

Contents

1 Details of comparision methods **2**

2 Performance comparison under different sample distribution **4**

3 SHAP analysis **5**

1 Details of comparision methods

To evaluate the performance of DeepHeteroCDA, we compare our methods with twelve baselines: Support Vector Machine [1] (SVM), Random Forest [2] (RF), k-Nearest Neighbors [3] (KNN), XGBoost [4], AdaBoost [5], DPMGCDA [6], MNGACDA [7], GATECDA [8], LAGCN [9], GCNMDA [10], VGAMF [11], VGAE [12].

- SVM is a supervised learning algorithm that finds the optimal hyperplane to separate data into different classes with the maximum margin.
- RF is an ensemble learning method that builds multiple decision trees and aggregates their predictions to improve accuracy and reduce overfitting.
- KNN is a simple, instance-based learning algorithm that classifies data based on the majority vote of its nearest neighbors.
- XGBoost is a powerful, efficient gradient boosting algorithm that builds decision trees sequentially, optimizing for both accuracy and computational speed.
- AdaBoost is an ensemble technique that adjusts the weight of misclassified instances to improve model performance by focusing on harder-to-classify examples.
- DPMGCDA employs a dual perspective learning approach combined with a path-masked graph autoencoder to predict circRNA–drug sensitivity associations. The model constructs circRNA–circRNA and drug–drug fusion similarity networks using similarity network fusion, ensuring a comprehensive integration of similarity information. It then builds a circRNA homogeneous graph, a drug homogeneous graph, and a circRNA–drug heterogeneous graph. From these graphs, DPMGCDA forms initial node features by leveraging both graph-level and feature-level perspectives.
- MNGACDA employs a multimodal network framework to integrate various sources of information related to circRNAs and drugs. Using a node-level attention mechanism within a graph auto-encoder (GAE), the model learns low-dimensional embeddings for both circRNAs and drugs. These learned embeddings are then fused through a convolutional neural network combiner module, and an inner product decoder is applied to predict potential circRNA–drug associations.

- GATECDA employs a Graph Attention Auto-Encoder (GATE) to extract low-dimensional representations of circRNAs and drugs. By constructing similarity networks, it assigns attention weights to node neighbors, capturing node-level features. These features are then used in a fully connected neural network to predict circRNA–drug sensitivity associations.

- LAGCN predicts drug–disease associations by integrating known associations into a heterogeneous network. It applies graph convolution to learn drug and disease embeddings and uses an attention mechanism to combine embeddings from multiple layers, scoring potential associations based on the integrated embeddings.

- GCNMDA uses a GCN-based framework to predict microbe–drug associations by constructing a heterogeneous network of microbe and drug similarities, along with microbe–drug interactions. It employs graph convolution, a CRF layer with attention for node similarity, and a random walk with restart to extract features.

- VGAMF predicts miRNA-disease associations by combining variational graph auto-encoder and matrix factorization. It generates non-linear representations from miRNA and disease similarity networks and linear representations from the miRNA-disease association matrix. A fully connected neural network then combines these representations to predict association scores.

- VGAE constructs an undirected, unweighted graph and learns low-dimensional embeddings of nodes using a two-layer Graph Convolutional Network (GCN) as the encoder. It employs a variational auto-encoder framework where latent node representations are learned and reconstructed using an inner product decoder, facilitating the prediction of graph links or associations.

2 Performance comparison under different sample distribution

To evaluate the performance of DeepHeteroCDA under different circRNA-drug sensitivity association scenarios, we have conducted additional experiments under different sample ratios (1:1, 1:2, 1:3, 1:4 and 1:5) and compared our method with DPMGCDA using five-fold cross-validation. The results are shown on Fig. S1. The experimental results indicate that as the positive-negative sample ratio increases, the area under the receiver operating characteristic curve (AUC) values of both DeepHeteroCDA and DPMGCDA remain stable without significant changes. In contrast, the Area Under the Precision-Recall Curve (AUPR) and F1 score exhibit noticeable declines. The stability of AUC can be attributed to its insensitivity to class distribution, as it primarily evaluates the model's ranking ability [13]. In comparison, AUPR and F1 score are more influenced by class imbalance, particularly when the proportion of positive samples decreases, which makes it challenging to maintain a balance between precision and recall. Across all tested sample ratios, DeepHeteroCDA consistently outperforms method DPMGCDA in all three metrics. Specifically, when the positive-negative sample ratio increases from 1:1 to 1:5, the AUPR of DeepHeteroCDA decreases by only 15.92%, while its F1 score drops by 17.04%. For DPMGCDA, the AUPR decreases by 23.25%, and its F1 score drops by 36.12%. These results demonstrate that method DeepHeteroCDA maintains robust performance across varying sample ratios. Consequently, DeepHeteroCDA is better suited for diverse practical application scenarios, showcasing its reliability in handling class-imbalanced data.

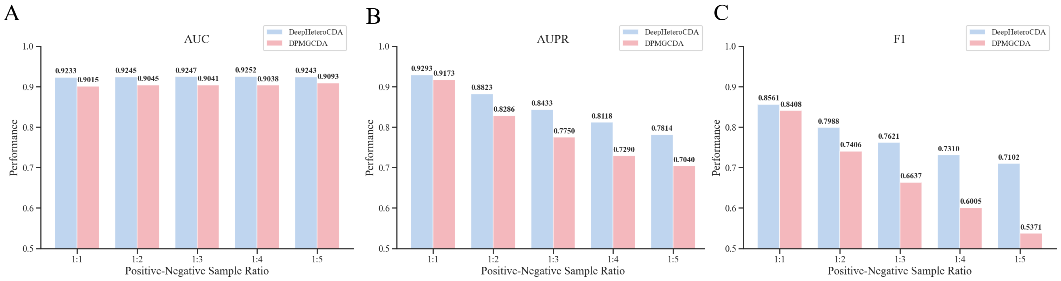

Figure S1: Performance comparison on three metrics under different positive-negative sample ratios. (A) AUC; (B) AUPR; (C) F1 score.

3 SHAP analysis

To improve the interpretability of our model, we conducted SHAP (SHapley Additive exPlanations) [14] analysis on all circRNA-drug samples in our dataset to enhance the interpretability of our model. This analysis helps identify the contribution of each feature to the prediction results, providing clearer insights into the key factors driving the model's prediction. A higher SHAP value indicates that the feature contributes more to predicting the positive class, while a lower SHAP value suggests that the feature contributes more to predicting the negative class. For each circRNA-drug sample in our dataset, we selected the results from the validation set in the five-fold cross-validation and performed SHAP analysis on four types of features in our model: circRNA comprehensive similarity matrix, drug comprehensive similarity matrix, association matrix, and drug representation matrix. The results are shown in Fig. S2. The left side of the plot displays the SHAP value analysis for positive samples in the dataset, while the right side shows the results for negative samples. We can observe that the SHAP values for the four features on the left are predominantly higher than those on the right, indicating that these features are effective in helping the model distinguish between positive and negative samples during the prediction process. The relatively extensive distribution of SHAP values for the four features across different samples indicates that variations in these feature values can exert different influences on predictions for different samples.

To further analyze the contribution of these four features to the model's prediction across the entire dataset, we present the boxplot of their SHAP values. As shown in Fig. S3, compared to drug comprehensive similarity matrix, the SHAP distribution of circRNA comprehensive similarity matrix is more balanced. This is because the features of drug in the model comprises two components: drug comprehensive similarity matrix and drug representation matrix. The SHAP values of drug comprehensive similarity matrix are skewed toward lower values, while those of feature drug representation matrix are skewed toward higher values. Together, they collectively contribute to the model's predictions as drug-related features. The difference in SHAP value distributions of these two features further demonstrates that our model learns the inter-feature relationships. Specifically, from the perspective of drugs, the prediction of positive samples may rely more on the specific structural properties of the drug, while the prediction of negative samples may be more dependent on the similar-

ity between drugs. Additionally, the relatively high SHAP values of association matrix indicate that the model can rely on existing association information to infer potential association relationships.

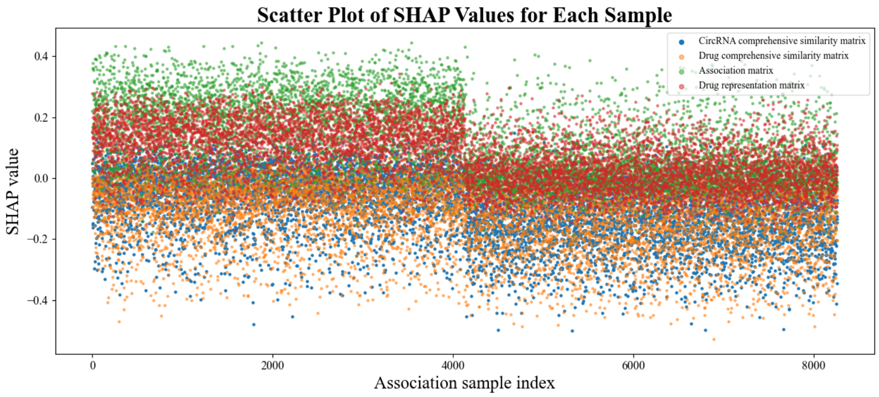

Figure S2: Scatter plot of SHAP values for each sample. The left side shows SHAP values for positive samples, and the right side for negative samples. Higher SHAP values indicate that the feature contributes more towards predicting the positive class, while lower values indicate a contribution towards predicting the negative class.

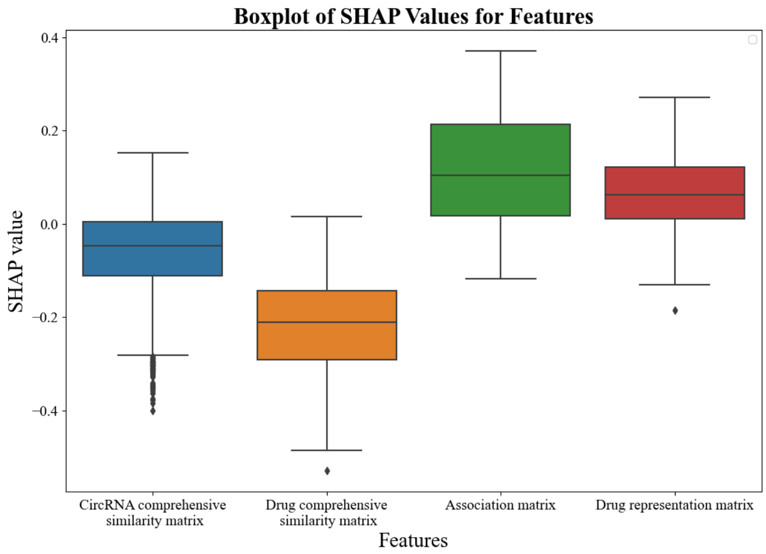

Figure S3: Boxplot of SHAP values for features. The plot illustrates the spread of SHAP values for each feature.

References

[1] Corinna Cortes and Vladimir Vapnik. Support-vector networks. *Machine learning*, 20:273–297, 1995.

[2] Leo Breiman. Random forests. *Machine learning*, 45:5–32, 2001.

[3] David W Aha, Dennis Kibler, and Marc K Albert. Instance-based learning algorithms. *Machine learning*, 6:37–66, 1991.

[4] Tianqi Chen and Carlos Guestrin. Xgboost: A scalable tree boosting system. In *Proceedings of the 22nd acm sigkdd international conference on knowledge discovery and data mining*, pages 785–794, 2016.

[5] Yoav Freund and Robert E Schapire. A decision-theoretic generalization of on-line learning and an application to boosting. *Journal of computer and system sciences*, 55(1):119–139, 1997.

[6] Yue Luo and Lei Deng. Dpmgcda: Deciphering circrna–drug sensitivity associations with dual perspective learning and path-masked graph autoencoder. *Journal of Chemical Information and Modeling*, 2024.

[7] Bo Yang and Hailin Chen. Predicting circrna-drug sensitivity associations by learning multimodal networks using graph auto-encoders and attention mechanism. *Briefings in Bioinformatics*, 24(1):bbac596, 2023.

[8] Lei Deng, Zixuan Liu, Yurong Qian, and Jingpu Zhang. Predicting circrna-drug sensitivity associations via graph attention auto-encoder. *BMC bioinformatics*, 23(1):1–15, 2022.

[9] Zhouxin Yu, Feng Huang, Xiaohan Zhao, Wenjie Xiao, and Wen Zhang. Predicting drug–disease associations through layer attention graph convolutional network. *Briefings in Bioinformatics*, 22(4):bbaa243, 2021.

[10] Yahui Long, Min Wu, Chee Keong Kwoh, Jiawei Luo, and Xiaoli Li. Predicting human microbe–drug associations via graph convolutional network with conditional random field. *Bioinformatics*, 36(19):4918–4927, 2020.

[11] Yulian Ding, Xiujuan Lei, Bo Liao, and Fang-Xiang Wu. Predicting mirna-disease associations based on multi-view variational graph auto-encoder with matrix factorization. *IEEE journal of biomedical and health informatics*, 26(1):446–457, 2021.

[12] Thomas N Kipf and Max Welling. Variational graph auto-encoders. *arXiv preprint arXiv:1611.07308*, 2016.

[13] Tom Fawcett. An introduction to roc analysis. *Pattern recognition letters*, 27(8):861–874, 2006.

[14] Scott Lundberg. A unified approach to interpreting model predictions. *arXiv preprint arXiv:1705.07874*, 2017.
